# Germline natural killer cell receptors modulating the T cell response

**DOI:** 10.3389/fimmu.2024.1477991

**Published:** 2024-11-04

**Authors:** Laura Mora-Bitria, Becca Asquith

**Affiliations:** Department of Infectious Disease, Faculty of Medicine, Imperial College London, London, United Kingdom

**Keywords:** NK cell regulation, T cell, T cell survival, innate receptor, NK cell

## Abstract

In addition to their central role during innate responses, NK cells regulate adaptive immunity through various mechanisms. A wide array of innate receptors has been involved in the NK cell regulatory function. However, the clinical implications of these regulatory pathways are poorly understood. Here, we review the experimental evidence on the effects of NK cells on T cells and their positive and negative consequences for disease outcome during T cell responses in humans.

## Introduction

The ability of a host to respond to pathogens in an improved fashion upon subsequent infections is a defence mechanism central to adaptive immunity. In jawed vertebrates, adaptive immunity is executed by T and B lymphocytes expressing a vast repertoire of somatically rearranged receptors. For example, the adult human body typically harbours 10^11^ T cells with 10^8^ different somatically generated T cell receptors (TCR) (1) out of the 10^20^ possible TCRs (2).

While this broad TCR repertoire can recognise the evolving pathogenic landscape, it can also erroneously respond to self-proteins. Furthermore, even when correctly responding to foreign antigen it is necessary to control the magnitude and longevity of the T cell response. Several control mechanisms modulate adaptive immunity. These vary from the deletion of clones with self-reactive TCRs to the regulation of the adaptive response by the innate immune system.

Here, we review the regulatory role of one particular component of innate immunity: natural killer (NK) cells and their germline-encoded receptors. We summarise the mechanisms by which NK cells have been shown to modulate T cells, the receptors and ligands involved, their expression patterns and evidence of their impact on human health.

## Multifaceted immune functions of NK cells

T, B cells and NK cells originate from a common lymphoid progenitor in the bone marrow. However, unlike T and B cells, NK cells do not express somatically rearranged receptors and are considered innate effectors. Upon activation, NK cells produce cytokines and execute a rapid cytotoxic function against infected or transformed cells. Although less than 20% of circulating lymphocytes are NK cells (3), their innate effector function is essential. Low NK cell cytotoxicity correlates with the risk of cancer (4), and more recent studies show that individuals with primary NK cell immunodeficiencies are more susceptible to herpesvirus infections and related morbidities (5). Besides these direct effects of NK cells on pathogen-infected and malignant cells, NK cells also modulate the adaptive response.

**Figure 1:**
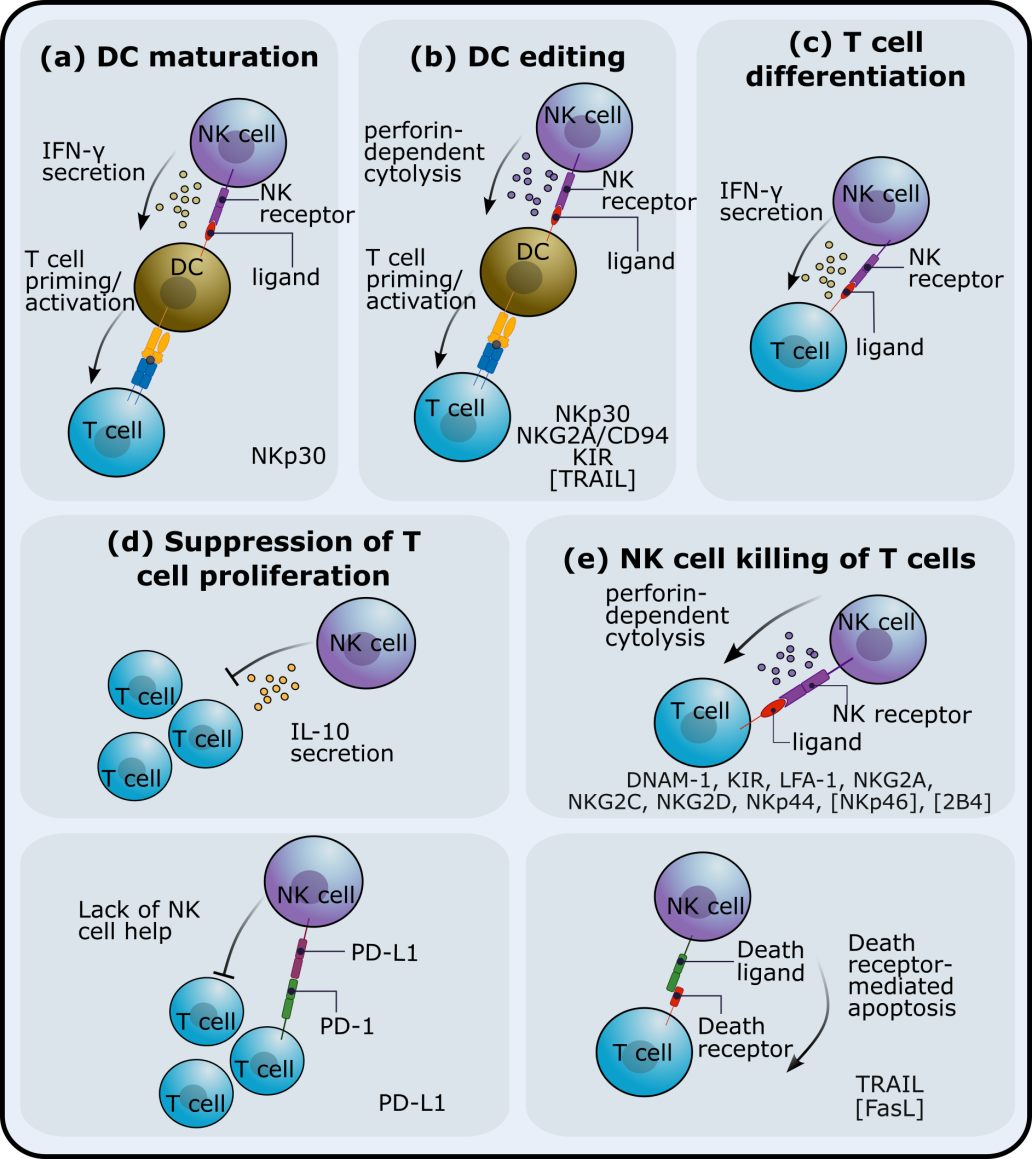
NK cell immunoregulatory mechanisms. NK cells can impact adaptive T cell responses through different pathways. NK cells can **(A)** affect dendritic cell (DC) maturation and/or **(B)** kill DCs and thus affect T cell priming. Interactions between NK cells and T cells have been shown to **(C)** promote T cell differentiation, **(D)** inhibit proliferation or expansion of T cells and **(E)** reduce T cell numbers via NK cell-mediated cytotoxicity. The receptors that have been implicated in these NK cell regulatory roles are listed at the bottom of each subplot. Receptors in square brackets have been studied in mice. The blue and yellow receptor-ligand complex in **(A, B)** indicates T-cell receptor (TCR) and major histocompatibility complex (MHC).

In healthy individuals, NK cells are found at low frequency in lymph nodes (3, 6), but *in vivo* studies in mice show that infection triggers NK cell recruitment to lymphoid tissue and their accumulation in T-cell rich sites (7, 8). Colocalisation of NK cells and T cells in lymph nodes – the sites of primary T cell activation – may facilitate communication between these two lymphocyte populations and influence the outcome of the immune response (7). Different mechanisms of NK cell regulation of T cells and how they impact adaptive responses have been described (**Figure 1**). To date, the impact of these NK cell regulatory pathways on the outcome of adaptive responses has been explored mainly in mouse models (**Table 1**). The human *in vitro* and/or ex vivo studies investigating NK immunoregulatory role are listed in **Table 2**.

**Table 1:** NK cell immunoregulation of T cell responses in mice.

Target population	NK cell- mediated mechanism	Effect on adaptive response	References	Model	T cell specificity
DCs	DC maturation	Enhancement of protective T cell responses	(9, 10)	Mouse *in vivo*	Unknown, tumour
		Inhibition of antitumour T cell responses	(11)	Mouse *in vivo, ex vivo, in vitro*	tumour antigen-specific
	DC elimination or “DC editing”	Impairment of T cell responses	(12)	Mouse *in vivo*, *ex vivo*, *In vitro*	MCMV
			(13)	Mouse *in vivo, ex vivo*	human papillomavirus, ovalbumin
		Enhancement of T cell responses	(14)	Mouse *in vivo*	Tumour specific
CD4^+^	CD4^+^ differentiation	Priming of T cells towards Th1	(15)	Mouse *in vivo*, *in vitro*	ovalbumin
	Suppression of CD4^+^ proliferation	Impaired antiviral T cell responses (liver)	(16)	Mouse *in vivo, ex vivo*, *in vitro*	LCMV
	CD4^+^ killing	Impairment of T and B antiviral responses	(17–19)	Mouse *in vivo*, *ex vivo, in vitro*	LCMV, MCMV
		Impairment of germinal center responses (affinity maturation)	(20)	Mouse *in vivo*	Hapten protein conjugate
		Prevention of immunopathology	(19)	Mouse *in vivo*	LCMV
		Prevention of autoimmunity	(18, 21, 22)	Mouse *in vivo, in vitro*	Type II collagen, myelin oligodendrocyte glycoprotein (MOG) MCMV
		Impairment of immune memory formation	(17)	Mouse *in vivo*, *ex vivo, in vitro*	LCMV
CD8^+^	CD8^+^ killing	Impairment of immune memory formation	(17, 23)	Mouse *in vivo*, *ex vivo, in vitro*	LCMV ovalbumin
		Impairment of antiviral T cell responses	(24)	Mouse *in vivo, in vitro*	LCMV
		Prevention of immunopathology	(25)	Mouse *in vivo*	LCMV
	Suppression of CD8^+^ proliferation/expansion	Impairment of antiviral and vaccine-induced antiviral response	(16, 26)	Mouse *in vivo, ex vivo*, *in vitro*	Vaccine- induced (ChAdOx), LCMV

Due to the size of the literature, only a sample of studies are reported for each mechanism. Note that the same NK cell immunoregulatory role can have both positive and negative effects on adaptive T cell responses.

**Table 2:** NK cell immunoregulation of T cell responses in humans.

Target population	NK cell- mediated mechanism	Effect on adaptive response	References	Model	T cell specificity
DCs	DC maturation	Priming of T cells towards Th1	(27)	Human *in vitro*	Not investigated
	DC elimination or “DC editing”	Unknown	(28–31)	Human *in vitro*	Not investigated
CD4^+^	CD4^+^ differentiation	Priming of T cells towards Th1	(32)	Human *in vitro*	Not investigated
	Suppression CD4^+^ proliferation	Unknown	(33)	Human *in vitro*	Bee venom allergen, phospholipase A2 (PLA) and *M. bovis*
			(34)	Human *in vitro*	Not investigated
	CD4^+^ killing	Unknown	(35–37)	Human *in vitro*	*M. bovis*, LPS
		Prevent autoreactivity	(38)	Human *in vitro, ex vivo*	Not investigated
CD8^+^	CD8^+^ killing	Increase in viral load	(39)	Human *in vitro, ex vivo*	HBV
		Decreased CD8^+^ clonal expansion	(40)	Human *in vitro, ex vivo*	HCMV
	Suppression of CD8^+^ expansion	Impairment of vaccine-induced antiviral response	(26)	Human *in vitro, ex vivo*	HBV

NK cells can impact T cell responses through interactions with other innate cells that in turn affect T cells (i.e. involving one or more additional cell populations) or with T cells. IFN-γ-producing NK cells can drive dendritic cell (DC) maturation, which in turn, prime Th1 T cell responses (27). This NK cell-mediated Th1 polarisation is dependent on the cytokine conditioning regime of NK cells and on cell-cell interactions between NK cells and DCs. NK cells can also kill DCs (29–31), a process known as DC editing as NK cells preferentially target immature DCs. DC editing might preferentially select more immunogenic DCs and has been shown to lead to more protective antitumour T cell responses in mice (14). However, NK cell regulation of DCs has also been shown to reduce T cell priming in a mouse tumour model (11) and to limit murine antiviral T cell responses, promoting, in this case, viral persistence (12). In a mouse model of allogeneic skin graft rejection, NK cells kill allogeneic DCs in graft draining lymph nodes, preventing alloreactive T cell responses (41).

NK cells can also interact with both CD4^+^ and CD8^+^ T cells. IFN-γ production by NK cells has been shown to promote the priming of Th1 T cell responses in lymph nodes, both in mice (15) and in humans (32). NK cells can also suppress T cell proliferation both in humans and in mice, an effect that is cell-cell contact dependent (16, 33). On the other hand, (42) showed that NK cells can kill CD4^+^ and CD8^+^ T cells in a perforin-dependent manner, an observation that has been validated by an increasing number of studies (reviewed in (43–45)).

NK-cell killing of T cells has been shown to impair both B and T cell responses in mice (17, 19, 20). In some contexts, e.g. during murine cytomegalovirus (MCMV) or lymphocytic choriomeningitis virus (LCMV) infections, NK cell-mediated suppression of the adaptive T cell response can be beneficial and prevent immunopathology (19, 46). Additionally, NK cells can act in synergy with Tregs, ensuring tolerance and eliminating incorrectly activated T cells that can cause autoimmunity (18, 21, 22, 47).

In the next section, we review the receptor-ligand pairs that have been reported to be involved in these NK cell regulatory functions to date.

## Receptors modulating NK cell regulation of T cells

NK cell effector functions, both cytolytic and cytokine production, are modulated by a wide array of activating and inhibitory receptors expressed by NK cells. These functions are typically correlated with the two NK cell subsets; the CD56^dim^ population is associated with superior cytolytic function whereas CD56^bright^ cells are potent cytokine producers. Few studies have looked at the role of each NK cell subset during regulation of T cell responses but a comprehensive human *in vitro* study by (35) found that both CD56^dim^ and CD56^bright^ NK cells were able to kill activated T cells to a similar extent (though their response to cytokines differed).

Below, we review the receptor-ligand pairs that have been involved in the NK cell-mediated regulation of T cells in humans. Most studies focus on the effect of receptor engagement on NK cell cytotoxic function against T cells, either via direct release of lytic granules or via the induction of death receptor-mediated apoptosis. Note that most if not all the receptors reviewed below can also be expressed by T cells and affect T cell function or survival in a direct manner. Here we focus on the indirect T cell regulation by NK cell regulatory functions.

### Death receptors

#### TRAIL-TRAILRs

Like Fas ligand (FasL), TRAIL (also known as TNFSF10) belongs to the tumour necrosis factor (TNF) superfamily. Both FasL and TRAIL activate the extrinsic apoptosis pathway through engagement of surface death receptors. *In vitro* evidence in humans indicates that NK cells expressing TRAIL can eliminate hepatitis B virus (HBV)-specific CD8^+^ T cells with upregulated TRAIL death receptor 2 (TRAIL-R2), also known as TNFRSF10B or DR5 (39). Moreover, they showed CD8^+^ T cell expression of TRAIL-R2 correlates with HBV viral load. A similar study found that TRAIL+ NK cells target activated CD4^+^ T cells that upregulate TRAIL-R1 (also known as DR4 or TNFRSF10A) and TRAIL-R2 (35). Engagement of the death receptor Fas on T cells has also been implicated in NK cell-mediated killing of CD4^+^ T cells in a mouse model of graft-versus-host disease (48). Whether Fas-FasL interactions result in NK cell-mediated T cell apoptosis has not been investigated in humans. Like Fas and TRAIL, the co-stimulatory molecule OX40 (TNFRSF4) also belongs to the TNF receptor superfamily and has also been implicated in NK cell regulation; there is some evidence that engagement of OX40 on T cells by OX40L+ NK cells promotes CD4^+^ T cell proliferation in humans *in vitro* (49).

### Activating receptors

#### NKG2D-MICA/B

NKG2D is distant member of the C-type lectin receptor NKG2 family, displaying limited homology with the other 6 NKG2 members. The NKG2 family contains both activating and inhibitory isoforms most of which form heterodimers with the CD94 molecule. An exception is NKG2D, which does not interact with CD94 and instead forms homodimers that engage the major histocompatibility complex class I chain-related proteins A and B (MICA/B) and the UL16 binding proteins (ULBP) as ligands. NKG2D is expressed on NK cells but also in CD8^+^, γδ T cells and a subset of CD4^+^ T cells. (42) showed that activated T cells are recognized and killed by NK cells in a perforin-dependent manner in mice. Activated T cells were also shown to upregulate NKG2D ligands. Moreover, staining intensity of NKG2D ligands on T cells correlated with susceptibility to NK cell lysis. Consistent with this, addition of anti-NKG2D or anti-NKG2D-ligand antibodies abrogated NK cell lysis. MICA and ULBP1-3 molecules are also upregulated in human CD4^+^ and CD8^+^ T cells undergoing proliferation upon stimulation with different antigens (50) whereas *M. tuberculosis*-expanded Tregs upregulate ULBP1 but not MICA/B molecules (36). NKG2D ligand expression renders activated CD4^+^, CD8^+^ and expanded Treg cells susceptible to NK cell cytolysis in an NKG2D dependent manner (36, 50). In line with these findings, a more recent study found that human CD4^+^ T cells activated with anti-CD3 and anti-CD28 coated beads are susceptible to NK cell degranulation. NK cell-mediated killing of T cells was dependent on engagement of multiple NK cell receptors, including NKG2D (35).

#### NKG2C/CD94-HLA-E

Like NKG2D, NKG2C is a NKG2 C-type lectin receptor but unlike NKG2D, NKG2C forms a heterodimer with CD94. NKG2C/CD94 heterodimer delivers activating signals when bound to its ligand, the HLA-E molecule. HLA-E presents a highly conserved nonamer peptide derived from the leader sequence of classical MHC class I molecules. Interestingly, the human cytomegalovirus (HCMV) UL40 signal peptide is homologous to the HLA-I leader peptide (51). HLA-E presentation of UL40-derived peptides is recognized by NKG2C+ NK cells, driving adaptive-like NK cell responses (52). However, HLA-E can also bind the inhibitory receptor NKG2A and thus can be used as an escape mechanism by HCMV. NKG2C expression is upregulated in NK cells from human cytomegalovirus (HCMV) infected individuals and has been implicated in innate NK cell control of HCMV infection (53). However, a recent study indicates that NKG2C+ NK cells can also regulate the HCMV-specific T cell response as NKG2C+ NK cells can kill HCMV-activated CD8^+^ T cells *in vitro* (54). In addition, NK cell cytotoxicity correlated with HLA-E expression, which is upregulated upon T cell activation and also positively correlates with T cell differentiation state.

#### NKp44-HLA-DP

The NKp44 receptor, also known as natural cytotoxicity receptor 2 (NCR2), belongs to the natural cytotoxicity receptor (NCR) family along with NKp46 (NCR1) and NKp30 (NCR3). NKp44 has multiple ligands including the extracellular matrix protein Nidogen-1 (55), the cancer-associated protein PCNA (56) and the soluble factor PDGF-DD (57). Additionally, NKp44 binds a subset of HLA-DP allotypes, including HLA-DP401 (58). NK cell recognition via NKp44 engagement was shown to be modulated by HLA-DP-presented peptides and resulted in NK cell degranulation. Since HLA-DP molecules are primarily expressed on antigen-presenting cells including B cells, NKp44-HLA-DP interactions could influence T cell responses via the NK cell interaction with a third immune cell population (58). However, a recent study reports upregulation of HLA-DP molecule in effector CD8^+^ T cells, especially in HCMV+ individuals (40). In addition, HLA-DP^+^ T cell frequencies were lower in individuals carrying the HLA-DP alleles coding for NKp44 ligands, suggesting that NKp44-HLA-DP engagement triggers NK cell suppression of T cells in a more direct manner.

#### aKIRs-HLA-I

Killer-cell immunoglobulin-like receptors are encoded by a polymorphic and polygenic gene family that contains both activating and inhibitory isoforms. KIRs are expressed on NK cells and a subset of terminally differentiated T cells. In NK cells, KIRs regulate functional maturation and innate effector function of NK cells through interactions with their ligands, which include the polymorphic HLA-I molecules. Like NKp44, KIRs do not recognise all HLA-I allotypes. In fact, each KIR binds to a set of HLA-I allotypes. Although the ligands for inhibitory KIRs (iKIRs) are well-established, the ligands for activating KIRs (aKIRs) are less well-defined due to their enhanced peptide specificity, which makes ligand identification more problematic (59, 60). Some aKIRs have been shown to bind HLA class I molecules in a similar fashion to their paired inhibitory KIR, albeit with higher peptide specificity (61). Peptide-specificity of aKIRs together with the high inter-individual variation at both KIR and HLA-I genes might explain the paucity of experimental studies investigating the role of aKIR-HLA interactions on NK cell killing of T cells. Nonetheless, there is some evidence that KIR2DS1-C2 interactions activate NK cell alloreactivity against DCs and phytohemagglutinin-induced T cell blasts (62), an effect that was abrogated by KIR2DS1 masking and modulated by expression of iKIRs.

#### DNAM-1-PVR/Nectin2

DNAM-1 (CD226) is an activating receptor expressed in different immune cell types including NK cells that binds the Nectin/Nectin-like family of adhesion molecules, including the poliovirus receptor (PVR) (also known as CD155) and Nectin2 (also known as CD112 or PVRL2). It has been shown that upon superantigen stimulation, T cells upregulate DNAM-1 ligand expression. Moreover, DNAM-1-PVR interactions modulated NK cell killing of activated and proliferating T cells (63). Interestingly, an inhibitory receptor that belongs to the same receptor family as DNAM-1, TIGIT, also binds PVR and Nectin2 as ligands and so DNAM-1 and TIGIT are considered paired receptors. To our knowledge, whether TIGIT affects NK cell regulatory function has not been studied.

### Inhibitory receptors

#### NKG2A/CD94-HLA-E

Like the NKG2C/CD94 heterodimer, NKG2A/CD94 binds HLA-E molecules as ligands but unlike NKG2C/CD94, HLA-E engagement results in inhibition of NK cell function. NKG2A/CD94-HLA-E interactions have also been implicated in NK cell regulation of T cells. *In vitro* experiments by (37) showed that NK cells inhibited CD4^+^ T cell proliferation by killing antigen-activated CD4^+^ T cells. The extent of NK cell killing was dependent on the degree of HLA-E expression on T cells, which in turn depended on the stimulatory conditions and the interaction with its receptor, NKG2A/CD94. A similar study confirmed that the HLA-E-NKG2A interaction protected activated T cells from NK cell-killing and that blocking the interaction could increase NK cell degranulation by up to two-fold (35). Finally, studies in mice show that the HLA-E functional homologue, Qa-1b, inhibits NK cell regulation of T cell responses (64). Qa-1b is upregulated in response to type I IFN and so IFN signalling protects activated T cells from NK cell elimination (65, 66). Together with TCR activation, type I IFN also induces HLA-E upregulation in human antigen-activated T cells (37), suggesting that the stimulation conditions influence the extent of NK cell elimination of T cells.

#### iKIRs-HLA-I

Similar to aKIRs, the impact of iKIR-HLA-I interactions on NK cell regulation of T cells remains poorly understood. There is some evidence that inhibitory signals driven by KIR3DL1-HLA-Bw4 or KIR2DL2/L3-HLA-C1 interactions inhibit NK cell cytotoxicity against T cell blasts in humans *in vitro*, even in the presence of KIR2DS1-C2 activating signals (62). Indirect evidence of a regulatory role of inhibitory receptors recognising MHC-I on NK cells (Ly49 receptors) can also be found in studies in mice, where lower levels of MHC-I molecules, either due to lack of IFN I signalling or due to deficiency of a transcriptional regulator of MHC-I genes, result in increased NK cell-mediated elimination of T cells (66, 67). In a more recent study using isotope labelling, we have shown that the number of iKIR-ligand gene pairs in an individual positively correlates with CD8^+^ T cell lifespan in humans *in vivo* (68); each additional iKIR-ligand gene pair increased T cell lifespan by 60 days. This effect was independent of iKIR expression on CD8^+^ T cells, suggesting that an indirect mechanism – possibly through NK cell regulation of T cells – is at play.

#### PD-1-PD-L1

It is well established that PD-L1 ligation of the “checkpoint receptor” PD-1 hampers TCR activation and acquisition of T cell effector function with some evidence indicating that PD-1 engagement can also decrease T cell survival. A recent study indicates that, in a murine model of chronic Hepatitis B virus infection, engagement of PD-1 on ChAdOx vaccine-induced T cells by PD-L1 on NK cells reduces the magnitude of the vaccine-induced T cell response; an effect that is reversed by NK cell depletion prior to vaccination (26). Consistent with this, a study in mice has shown that liver resident PD-L1+ NK cells suppress proliferation of both CD4^+^ and CD8^+^ T cells upon PD-1 engagement, impairing antiviral T cell responses *in vivo* (16). This observation has been recapitulated in humans *in vitro*. In this case, activated T cells displayed reduced proliferation when co-cultured with tumour experienced PD-L1+ NK cells, an effect that was not observed when T cells were co-cultured with non-tumour experienced NK cells. Moreover, the negative effect on T cell proliferation by PD-L1+ NK cells was reversed by anti-PD-L1 blockade (69). Human *in vitro* experiments suggest that PD-L1 engagement reduces NK-cell IFN-γ secretion, providing a plausible mechanism underlying the PD-L1 dependent suppression of T cell expansion (26). Finally, the PD-L1/PD-1 axis has also been implicated in NK cell-mediated regulation of DC maturation. In this study, depletion of NK cells increased DC maturation and enhanced priming and recall antitumour T cell responses in mice (11).

## Variation in NK cell control of T cell responses

**Figure 2:**
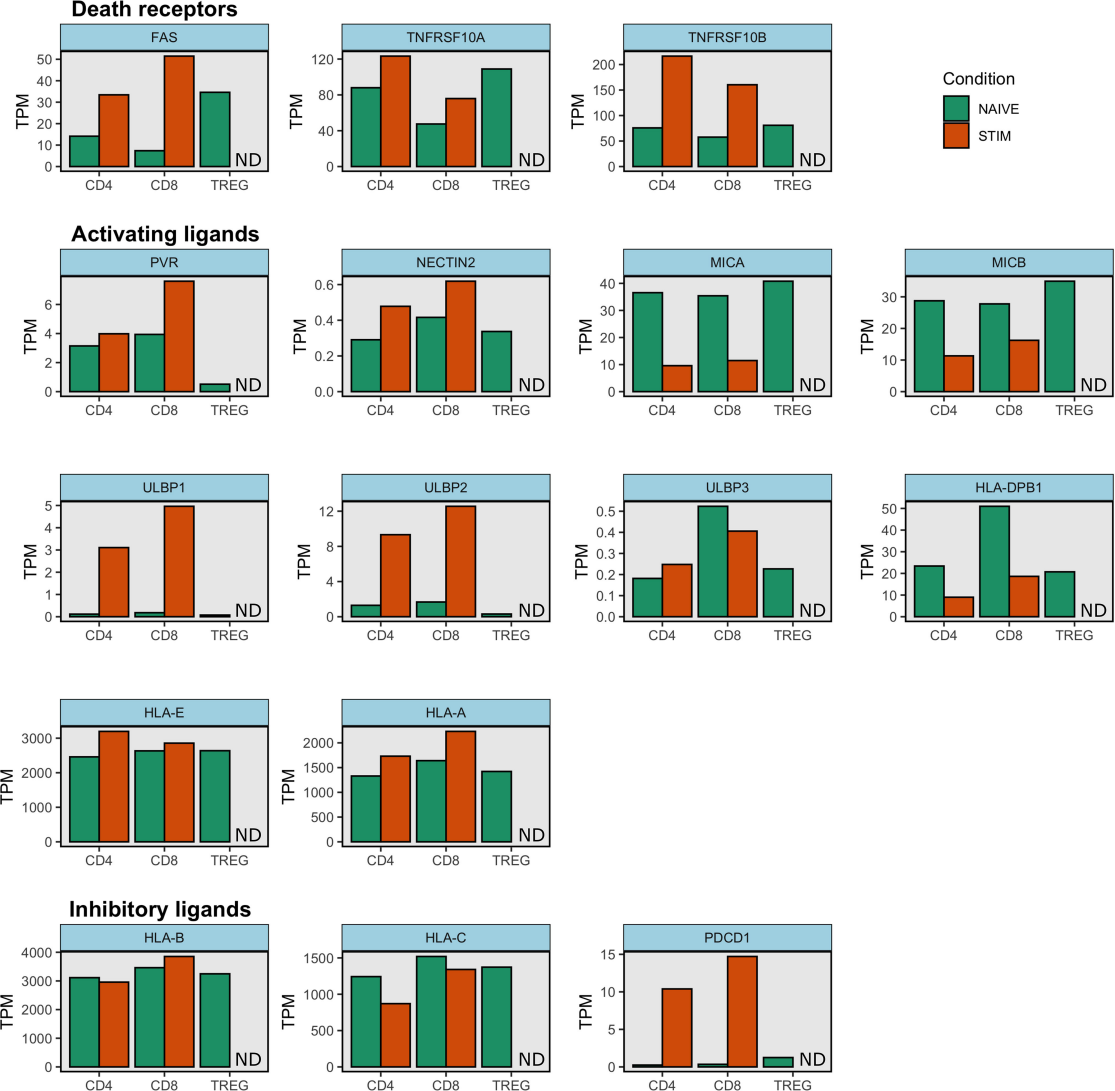
NK cell ligand expression on stimulated and unstimulated naïve T cells. Gene expression data from Schmiedel et al. was downloaded from the Human Protein Atlas (71). This dataset contains transcript expression levels per gene in 15 immune cell types. Mean transcripts per million (TPM) are shown for unstimulated and stimulated CD4^+^ and CD8^+^ naïve cell types as well as for unstimulated naïve Treg cells. Stimulated naïve Treg condition was not tested and is indicated as ND (Not Done) in each subplot. Gene expression was measured with RNA sequencing. Samples were stimulated with anti-CD3/CD28 beads for 4h (70). Note HLA-A and HLA-E molecules also bind inhibitory receptors and so they can function as inhibitory ligands. Similarly some HLA-B and HLA-C molecules also engage activating receptors.

Activating and inhibitory ligands for NK cell receptors are upregulated upon T cell activation, either by TCR ligation or cytokine stimulation. Therefore, it is likely that activated T cells are the primary targets of NK cell regulation (44). Indeed, by looking at gene expression data from (70) we find a consistent pattern with both naïve CD4^+^ and naïve CD8^+^ T cells upregulating the expression of death receptors FAS and TRAIL-R (1 and 2) upon activation (**Figure 2**). Therefore, NK cells expressing death receptor ligands might regulate recently activated T cells. In the case of ligands for NK activating receptors the picture is more mixed. HLA-E and HLA-A expression increases upon activation and although expression levels are lower, ULBP1-2 (but not ULBP3), Nectin2 and PVR are also upregulated upon activation. Surprisingly, MICA/B are downregulated in activated naïve T cells and HLA-DPB1 follows a similar pattern. Regarding inhibitory ligands, expression of HLA-C decreases in both CD4^+^ and CD8^+^ naïve T cells. However, HLA-B expression is higher in activated CD8^+^ T cells and remains constant in naïve activated CD4^+^ T cells compared to unstimulated samples. It is worth noting that these samples were stimulated with anti-CD3/CD28 beads for 4h (70) and so it is possible that if cells were stimulated for longer or under different conditions (e.g. with cytokines) we would observe different expression patterns. Additionally, different T cell subsets might upregulate different ligands so future studies might clarify if, upon stimulation, populations like naïve Tregs or memory T cells follow the same or different ligand expression patterns as conventional naïve T cells.

Context (together with variable expression of inhibitory and activating receptors/ligands) seem to affect the extent of NK cell regulation. For example, TRAIL has been described in the elimination of T cells *in vitro* (35, 39), but not in mice *in vivo* (16, 19). In LCMV infection, NK cells in mice kill activated CD4^+^ T cells (19) and follicular helper T (Tfh) cells (17) but the opposite happens during vaccinia virus infection in mice, where NK cells display poor cytotoxicity towards activated CD4^+^ T cells (72). This poor cytotoxicity is probably due to elevated expression of the inhibitory receptor NKG2A (72). Similarly, while in protein-immunized mice NK cells restrain germinal center responses (20), mice with previous LCMV vaccination had protective recall T cell responses against LCMV challenge (73). The authors suggest that vaccine-elicited T cell responses are not subjected to NK cell regulation (73) but this is in direct contrast with work from Diniz et al. showing NK regulation of responses induced by the ChAdOx vaccine.

Besides stimulation conditions, another factor that might affect the NK cell regulatory function is genetic variation. Some of the receptors and ligands described above, like KIRs and classical HLA molecules, display the highest levels of polymorphism in the human genome; only certain ligand alleles encode proteins that will bind the receptor and vice versa. Therefore, NK cell regulation is not only shaped by the array of receptor-ligand pairs expressed at a given time and context but also by the genetic makeup of the individual.

One the most polymorphic and polygenic NK cell receptor families is the KIR family. Individuals vary in the number of activating and inhibitory KIRs they carry in their genome. Furthermore, this gene content variation is enhanced by allelic diversity at each KIR. KIR alleles can affect protein expression and binding strength (74–76). Therefore, KIR diversity might impact NK cell regulatory function and determine the outcome of T cell responses.

A third level of diversity is conferred by KIR ligands. Each KIR binds a subset of ligands with similar if not higher levels of polymorphism, the HLA-I molecules. iKIRs bind HLA-I molecules in broad allele groups i.e. KIR2DL1 binds HLA-C2 allotypes whereas KIR2DL2/L3 bind C1 bearing molecules, with weak binding to C2 also reported for KIR2DL2 (77, 78). KIR3DL1, on the other hand, binds HLA-A and HLA-B molecules with the Bw4 motif (79). Therefore, combinations between the highly polymorphic and unlinked KIR and HLA loci shape functional KIR polymorphism and we have recently reported that these genotype combinations might affect NK cell regulatory function. Specifically, we have shown that the number of iKIR-HLA receptor-ligand genes present in the genome affects T cell lifespan in humans *in vivo* (68). In addition, we showed that the number of iKIR-ligand pairs was associated with an aging CD57+ phenotype in both CD4^+^ and CD8^+^ T cell subsets consistent with the increase in T cell lifespan. This effect is not driven by T cells expressing iKIRs, indicating that another population expressing iKIRs, possibly NK cells, is responsible for modulating T cell lifespan, probably by one of the mechanisms discussed in the previous sections.

Another NK cell regulatory pathway that is affected by genetic polymorphism is NKG2A/CD94-HLA-E. Although NKG2A, CD94, and HLA-E are highly monomorphic, a polymorphic site in the HLA-B leader region determines whether HLA-B-derived leader peptides can bind to HLA-E or not and thus engage with NKG2A/CD94 (80). Consistent with this, NK cells from individuals carrying the HLA-B variant that generates functional peptides for HLA-E display higher degranulation capacity (81). To our knowledge, whether this polymorphism affects T cell survival has not been explored.

## Clinical relevance of NK cell modulation of T cell survival

As we have seen, genetic variation at the receptor and ligand loci might affect NK/T cell interactions and thus shape disease outcomes. We have immunogenetic evidence that this may be the case in chronic viral infections and type 1 diabetes [reviewed in (82)]. Briefly, we have found that the number of iKIR-HLA gene pairs enhances HLA class I associations in three different persistent viral infections (83, 84). Given that the 11 HLA class I associations studied are all thought to be T cell mediated (85), we suggest that this effect on HLA class I risk indicates that iKIRs affect HLA-restricted CD8^+^ T cell responses. On the contrary, iKIR-HLA gene pairs weaken the protective effect of HLA class II protective genotypes in T1D and thus contribute to T1D risk (86).

We speculate that inhibitory signals mediated by iKIR-HLA interactions dampen the NK cell regulatory effect and may explain the observed correlation between functional iKIR genotype and T cell survival (68). This experimental observation fits well with our immunogenetic findings; increased T lifespan has beneficial effects in the context of viral infections but exacerbates autoimmune T cell responses. Accordingly, dampening of NK cell regulation seems to affect human B cell responses as well; HIV-1-infected individuals that make broadly neutralising antibodies (bnAbs) have higher levels of dysfunctional NK cells compared to HIV-1 individuals that do not produce bnAbs (87).

It is worth noting that the HLA-B haplotype that delivers functional leader peptides for NKG2A recognition via HLA-E – the 21M haplotype – does not contain iKIR ligand genes (80). This MM genotype has been associated with improved outcome in acute myeloid leukaemia patients undergoing immunotherapy (81). The 21M variant has also been associated with faster HIV-1 seroconversion (88), suggesting that NKG2A-mediated NK cell inhibition or possible lack of iKIR inhibition influences immune responses. Whether the NK cell immunoregulatory role is the underlying mechanism of this effect has not been directly explored yet.

Finally, indirect immunogenetic evidence hinting at the relevance of NK/T cell interactions also comes from cancer patients undergoing immunotherapy. In the context of immune checkpoint blockade – where immune checkpoint inhibitors unleash the antitumour T cell response – two studies have found that KIRs predict overall survival and progression-free survival. Specifically, (89) found that in non-small cell lung cancer patients treated with anti-PD-L1 therapy, those with 2 functional iKIR genes had increased survival compared with those with one or no functional iKIRs. Along the same lines, carriage of the KIR3DS1 allele (activating KIR) was associated with worse progression-free survival in another non-small cell lung cancer study with patients treated with anti-PD-1 (90). Therefore, inhibition of NK cell function might prevent the elimination of activated T cells, promoting T cell survival and thus leading to more effective antitumour T cell responses.

## The role of innate receptor diversity during NK cell regulation of T cell responses

It’s unclear whether having multiple regulatory checkpoints represents redundancy which guarantees robust regulation, or if different inflammatory signals activate specific regulatory pathways – either positive or negative –, or if both factors are at play.

Redundancy in NK cell regulatory pathways might be a result of the evolutionary pressure of pathogens on the host immune system. To keep up with the ever-changing nature of pathogens, hosts need to constantly develop new immune defence mechanisms. Diversification of the innate germline-encoded receptors represents one strategy to ensure robust responses against pathogen subversion (91). These diverse innate pathways might have been subsequently co-opted for NK cell regulation of adaptive T cell responses. In this scenario, NK cell regulation can be viewed as *collateral damage* due to close proximity between NK cells and T cells in the sites of T cell activation (44). Therefore, T cells had multiple pathways available to escape NK cell effects through the expression of different ligands for NK cell receptors. A different explanation would be that the existence of a complex regulatory circuit was the pre-condition for the radical innovation of adaptive immunity to happen (92); robust peripheral regulatory mechanisms might have allowed safe (and controlled) exponential growth of somatically diversified immune responses.

Another plausible explanation is that the different receptor-ligand systems serve in different contexts (93, 94). Similarly, the existence of multiple signalling pathways might represent a cross-validation mechanism; regulation is activated or deactivated in the presence or absence of certain receptor-ligand interactions. So different T cell activation conditions like acute viral infection, autoimmunity or chronic inflammation, might trigger the expression of different NK receptor ligands on T cells. In turn, the pattern of ligand expression on T cells might determine the outcome of NK cell regulation.

## Conclusions

NK cells are one of the main effectors of the innate immune system. In addition, NK cells regulate adaptive T cell responses through multiple mechanisms (**Table 1**). Various NK cell receptors and their ligands have been found to be involved in this NK cell regulatory function (**Table 3**). Whilst there are a large number of important studies in mice and *in vitro*, the consequences of these regulatory effects for human health remain underexplored.

**Table 3:** Receptor-ligand pairs involved in NK cell regulation of T cells.

Receptor	Ligand
Death receptors	
FAS	**FASLG**
TNFRSF10A (DR4, TRAIL-R1) TNFRSF10B (DR5, TRAIL-R2)	**TNFSF10 (TRAIL)**
Activating receptors	
**KLRK1 (NKG2D)**	MICA MICB ULBP1-3
**KLRC2 (NKG2C)**	HLA-E
**CD226 (DNAM-1)**	PVR NECTIN2
**KIR2DS1**	HLA-C*
**KIR2DS2**	HLA-C*
**KIR2DS4**	HLA-A* HLA-C*
**KIR3DS1**	HLA-B*
**NCR2 (NKp44)**	HLA-DP^¨^
Inhibitory receptors	
**KLRC1 (NKG2A/CD94)**	HLA-E
**KIR2DL1**	HLA-C*
**KIR2DL2/L3**	HLA-C*
**KIR3DL1**	HLA-A* HLA-B*
PDCD1 (PD-1)	**CD274 (PD-L1, B7-H)**

Death, activating and inhibitory receptors that have been related to NK cell regulation of T cell responses in humans. Note that this list is a subset of the receptor-ligands that control NK cell function and those receptor-ligands interactions that have not been reported to participate in NK cell regulatory roles are not included e.g. KIR3DS1 and HLA-F interaction. Receptor and ligand symbols are reported according to HUGO gene nomenclature. Symbols in brackets are commonly used aliases. *KIR2DL1, KIR2DL2/L3 and KIR3DL1 recognise a subset of HLA-I molecules, namely C2, C1 and Bw4 allotypes respectively. This is also the case for the paired activating KIRs KIR2DS1, KIR2DS2 and KIR3DS1 albeit with higher peptide specificity. NCR2 also recognises a subset of HLA-DP molecules, namely HLA-DP401 allotypes. Receptors and ligands implicated in a NK cell regulatory function that are expressed on NK cells (for regulatory function) are showed in purple bold font. Conversely, corresponding ligands or receptors expressed on T cells (to acquit their regulatory function) are showed in blue normal font.
